# Trajectories of blood parameters and body weight during inpatient treatment of anorexia nervosa with extreme underweight

**DOI:** 10.1186/s40337-026-01734-3

**Published:** 2026-08-12

**Authors:** Eva P. Wuttke, Adrian Meule, Ulrich Cuntz, Ulrich Voderholzer

**Affiliations:** 1 Department of Psychiatry and Psychotherapy, LMU University Hospital, LMU Medizin, Ludwig-Maximilians-Universität München, Munich, Germany; 2 Schoen Clinic Roseneck, Prien am Chiemsee, Germany; 3 Department of Psychology, University of Regensburg, Regensburg, Germany; 4 Department of Medicine, HMU Health and Medical University, Düsseldorf, Germany; 5 PMU, Paracelsus Medical University Salzburg, Salzburg, Austria; 6 Department of Psychiatry and Psychotherapy, University Hospital of Freiburg, Freiburg, Germany

**Keywords:** Extreme anorexia nervosa, Laboratory parameters, Illness duration, Body mass index

## Abstract

**Background:**

Laboratory parameters and body weight are objective markers of physical stability in patients with severe and extreme anorexia nervosa. However, systematic analyses of trajectories during inpatient treatment with high-calorie refeeding and prophylactic phosphate supplementation remain limited in extremely underweight persons. Therefore, it is essential to gain a comprehensive understanding of changes in laboratory parameters, body mass index, and factors influencing these trajectories under this treatment approach in patients with severe and extreme anorexia nervosa, who are at particularly high risk of medical complications.

**Methods:**

Retrospective data of a six-week treatment period from 519 female inpatients of a specialized care unit with anorexia nervosa (body mass index at admission: *M* = 12.6 kg/m^2^, *SD* = 1.35) were analyzed. Laboratory parameters (hemoglobin, thrombocytes, leukocytes, aspartate aminotransferase, alanine aminotransferase, creatine kinase, sodium, potassium, and phosphate) and body weight were assessed weekly.

**Results:**

More than one-third of patients showed leukopenia or hypertransaminasemia at admission. Most laboratory values and body mass index tended to ameliorate during the six-week treatment period. Lower body mass index at admission or longer illness duration was associated with larger changes in body mass index and several laboratory parameters (e.g., hemoglobin or aspartate aminotransferase).

**Conclusions:**

These findings provide a detailed characterization of laboratory changes during inpatient refeeding with prophylactic phosphate supplementation. The observed temporal patterns of laboratory stabilization may help inform individualized monitoring strategies and provide a basis for evaluating whether the frequency of routine laboratory testing can be tailored over a longer course of treatment according to clinical status and resolution of previous abnormalities. Furthermore, these findings may support the effectiveness and safety of inpatient refeeding protocols with prophylactic phosphate supplementation in patients with severe and extreme anorexia nervosa and an extremely low body mass index at admission (< 13 kg/m^2^).

**Supplementary Information:**

The online version contains supplementary material available at 10.1186/s40337-026-01734-3.

## Abstract (plain language summary)

People with severe and extreme anorexia nervosa have very low body weight and often abnormal blood values, which are important signs of physical health. However, it is not well known how these values change during inpatient treatment with high-calorie nutrition and prophylactic phosphate supplementation. In this study, we looked at changes in body weight and blood test results over the first six weeks of treatment, as well as factors that might influence these changes. We analyzed data from 519 female patients. At the start of treatment, many patients had abnormal blood values. Over the six-week treatment period, most blood values improved, and patients gained weight. Patients with lower body weight or longer illness duration often showed greater changes. These findings improve our understanding of how blood values change during refeeding and may help clinicians decide how closely blood tests need to be monitored throughout treatment. They may also support the effectiveness and safety of inpatient high-calorie treatment with prophylactic phosphate supplementation, even for patients with very low body weight.

**Supplementary Information:**

The online version contains supplementary material available at 10.1186/s40337-026-01734-3.

## Introduction

Anorexia nervosa (AN) is a severe mental disorder with the second highest mortality rate among all mental disorders [1]. It is characterized by a significantly low body weight maintained through behaviors such as restrictive eating, purging (e.g., self-induced vomiting or misuse of laxatives), and excessive exercise, which are driven by an intense fear of weight gain [2–4]. According to the current version of the Diagnostic and Statistical Manual of Mental Disorders (DSM–5), a body mass index (BMI) below 15 kg/m^2^ is classified as extreme, indicating pronounced medical vulnerability [5]. Approximately one quarter of persons with AN develop a long-lasting course, with severe and enduring presentations characterized by prolonged illness duration, repeated unsuccessful treatments, and psychological distress [6–8]. Due to the significant medical and psychological burden of AN, inpatient treatment is required in approximately one third of patients according to a recent meta-analysis, especially when psychiatric comorbidity, the need for close supervision, or medical instability is present [6, 9]. This underlines the need for a better understanding of the mechanisms underlying stabilization in AN.

Laboratory parameters and body weight provide easily accessible and objective markers of physical stability in patients with extreme AN. Previous research has reported a variety of biochemical abnormalities associated with starvation in AN [10, 11]. Studies from specialized medical stabilization units in patients with extreme AN (BMI < 15 kg/m^2^) have reported high rates of medical complications [12, 13]. Hemoglobin levels can be reduced [10], contributing to fatigue, cognitive impairment, and depressive symptoms [14]. Thrombocyte counts are sometimes reduced due to bone marrow suppression [10]. Nearly one-third of patients showed leukopenia in the acute illness stage [15]. Younger persons and those with a lower BMI tend to have the highest incidence of these cytopenias [16]. Liver enzymes, such as alanine aminotransferase (ALT) and the less-specific enzyme aspartate aminotransferase (AST), are often elevated [17]. Elevated creatine kinase (CK) levels have been reported in AN, often reflecting excessive physical activity [18]. Electrolyte disturbances, particularly hypokalemia and hyponatremia, are common, especially in persons with purging behavior [10]. Although phosphate levels may appear normal in the acute starvation period, they can drop sharply during refeeding, potentially leading to life-threatening complications [19]. Although these studies provide important insights into medical complications and aspects of the early treatment course, they have primarily focused on the prevalence and occurrence of complications rather than systematically examining longitudinal trajectories of laboratory parameters during inpatient treatment. In particular, longitudinal data spanning the early weeks of nutritional rehabilitation with routine prophylactic phosphate supplementation in large cohorts of patients with extreme AN remain scarce.

Given these medical complications at admission, nutritional rehabilitation is a central component of inpatient treatment. During early inpatient treatment, rapid weight gain is typically required to restore medical stability. However, this process carries the risk of refeeding syndrome, particularly hypophosphatemia [20]. At the same time, excessive caloric restriction during refeeding may lead to underfeeding syndrome, which is associated with delayed weight restoration, prolonged medical instability, and longer hospital stays [21]. Accordingly, higher-calorie refeeding protocols are increasingly regarded as the standard of care for inpatient stabilization and can be safely implemented under close medical monitoring [22, 23].

Beyond the safety of refeeding protocols, understanding the pattern of weight restoration during inpatient treatment is also clinically relevant. Previous results suggest that weight trajectories during inpatient treatment were non-linear, with rapid increases at the beginning and a deceleration over time [24]. Several clinical and psychological factors have been identified as moderators of weight restoration. Discharge BMI itself was a strong predictor of weight status at follow-up, independent of illness chronicity [25]. Furthermore, patients with longer illness duration gained weight at similar rates during inpatient treatment and showed comparable short-term outcomes to those with shorter illness duration [25]. Using duration of amenorrhea as a proxy for illness duration, De Filippo, Marra [26] found associations with neutropenia, anemia, and thrombocytopenia. Similarly, longer illness duration has been associated with lower potassium and calcium levels as well as other biochemical abnormalities despite comparable BMI [27]. Nevertheless, the physiological relevance of illness duration remains uncertain, as it does not necessarily reflect the duration of severity of undernutrition. Consequently, whether illness duration influences trajectories of laboratory parameters during inpatient treatment remains an open question. Moreover, evidence regarding the longitudinal effects of BMI and illness duration in large samples of extremely underweight patients remains limited.

Building on this gap, the aim of this retrospective study was threefold. First, we aimed to quantify the proportion of patients presenting laboratory values outside the reference range at admission and to examine how these rates changed from admission to week 6. Second, we investigated the trajectories of BMI and central laboratory parameters during six weeks of inpatient treatment with high-calorie refeeding and phosphate supplementation using robust linear mixed models. We expected that BMI would increase over time and that the laboratory parameters would normalize during treatment. Third, we examined whether these trajectories varied as a function of baseline BMI. In addition, we exploratorily investigated whether illness duration was associated with differences in these trajectories.

## Methods

### Sample characteristics

According to the ethics committee at LMU Munich, retrospective analyses of completely anonymized data are exempt from requiring ethical approval or signed informed consent. At the Schoen Clinic Roseneck (Prien am Chiemsee, Germany), data from diagnostic assessments (e.g., age, body height, body weight, length of stay, diagnoses, and blood values) are automatically transferred to a database from which they can be exported without any identifying information (e.g., name, date of birth, and place of residence) by authorized employees. Thus, accessing individual patient charts was not necessary.

Data of 519 women with AN or atypical AN according to the ICD-10 criteria (ICD–10 codes F50.0 [95.4%, *n* = 495] or F50.1 [4.60%, *n* = 24]) who were treated in an inpatient specialized care unit at Schoen Clinic Roseneck between 2016 and 2023 were analyzed. Information on AN subtype (restricting versus binge/purging subtype) was not available in the database used for the present analyses. Inclusion was restricted to persons with an admission BMI < 15.00 kg/m^2^, corresponding to the extreme severity range according to the DSM–5 [5]. Mean age was 28.5 years (*SD* = 10.8, range = 18–65). Mean illness duration was 10.6 years (available for *n* = 273, *SD* = 9.74, range = 1–45). At admission, mean body weight was 34.2 kg (*SD* = 4.80, range = 22.4–49.2), corresponding to a mean BMI of 12.6 kg/m² (*SD* = 1.35, range = 9.00–14.99). More than half of the sample (62.6%, *n* = 325) had at least one comorbid mental disorder, the most common of which were affective disorders (ICD–10 code F3: *n* = 150, 28.9%) and anxiety disorders (ICD–10 code F4: *n* = 153, 29.5%). Mean length of stay was *M* = 81.4 days (*SD* = 58.6, range = 2–289 days).

Treatment at the specialized inpatient unit for patients with extreme underweight (BMI < 15 kg/m²) and/or excessive purging or exercising was delivered in accordance with the German S3-guidelines for the treatment of AN [9]. No coercive treatment was administered. Following medical stabilization, patients typically continued treatment (over six weeks) within specialized eating disorder services at the Schoen Clinic Roseneck and stepped-care treatment pathways according to their individual clinical needs. All inpatients underwent a standardized, multimodal treatment program based on cognitive-behavioral therapy, comprising individual psychotherapysessions, group therapy sessions, meal preparation classes, body image exposure, nutrition counseling, food intake protocols, and clinical management of medical complications. Nutritional rehabilitation was initiated on the first day of admission using a high-calorie refeeding protocol, aiming for a minimum weekly weight gain of 0.70–1.00 kg. All patients initially received a standardized non-vegetarian or vegetarian meal plan providing approximately 2000 kcal/day, consisting of three orally administered main meals per day.

If patients consumed more than 50% but not all of a meal, they were required to drink one oral nutritional supplement (Fresubin^®^, 400 kcal). If less than 50% was consumed, two supplements were provided to compensate for the missed calorie intake. Meals were consumed in a structured therapeutic group setting under staff supervision, with post-meal monitoring to minimize compensatory behaviors. In cases requiring additional support, one-to-one supervision was provided. Consequently, actual caloric intake was assumed to closely approximate prescribed caloric intake, although objective measurements of individual intake were not available. Nasogastric tube feeding was rarely required in clinical practice and, when necessary, was used only temporarily (approximately 0–2% of cases). However, its exact frequency in the present sample was not systematically recorded. Some patients were admitted with nasogastric feeding already in place, the nasogastric tube was normally removed within the first few days after admission. The rare cases requiring tube feeding were limited to patients with persistently insufficient oral intake despite intensive therapeutic support and meal supervision. The therapeutic approach prioritized the normalization of oral eating behavior whenever possible.

During treatment, meal plans were individually adjusted based on weight trajectories, aiming for a minimum weekly weight gain of 0,7 kg and clinical judgment, without a standardized protocol for caloric advancement. Most patients achieved sufficient initial weight gain with the starting meal plan of about 2000 kcal/day and strict restriction of physical activity, such that caloric increases were infrequently required during the first weeks of treatment. Nutritional intake was increased by enlarging meal proportions and/or adding snacks or oral nutritional supplements. As treatment progressed, meal plans typically provided around 2800 kcal/day, although substantial interindividual variation existed and prescriptions occasionally exceeded 4000 kcal/day. Importantly, caloric values were not routinely communicated to patients. Instead, modifications for the meal plan were described in terms of meal portions and eating patterns comparable to those of healthy individuals.

To prevent refeeding syndrome, electrolytes were monitored daily during the first week of treatment and subsequently at least weekly for four weeks, with further assessments performed according to clinical requirements. All patients received prophylactic phosphate supplementation (613 mg disodium hydrogen phosphate three times daily; Reducto^®^ special). Phosphate supplementation was generally continued for 28 days in patients with an admission BMI < 13 kg/m^2^ and subsequently adjusted according to laboratory findings, with a target serum phosphate concentration of ≥ 1.5 mmol/L. In cases where serum phosphate levels decreased despite supplementation or where there were signs suggestive of emerging rhabdomyolysis, phosphate supplementation was increased. No patient discontinued phosphate supplementation because of adverse effects. In addition, patients received thiamine (200 mg/day) and vitamin B complex supplementation for the first 14 days of refeeding. Potassium and magnesium were supplemented according to laboratory findings.

Vital signs, including blood pressure, heart rate, and development of peripheral edema, were closely monitored through refeeding because fluid shifts and edema are common during the early phase of nutritional rehabilitation. Daily medical contacts were performed. Intravenous fluid replacement was used cautiously because rapid volume expansion may precipitate cardiac complications in severely malnourished patients; therefore circulatory adaptations were corrected gradually over several days. Diuretics were reserved for selected cases of clinically significant edema.

### Measures

*BMI.* Height was measured at admission, and weight was measured weekly without blinding to patients or staff. In case of critical underweight (BMI < 13.5 kg/m^2^), weight measurement was increased to three times per week, if clinically indicated, up to daily. However, for the present analyses, only weekly weight measurements were available and used. BMI was calculated as weight in kilograms divided by height in meters squared (kg/m²).

*Blood values.* Blood samples were routinely collected at admission and weekly during the inpatient stay. Electrolytes were monitored daily during the first week of treatment and subsequently at least weekly for four weeks, with further assessments performed according to clinical requirements. Not all patients had laboratory measurements available at every assessment. Some patients were discharged before the week 6 assessment. In addition, some patients were transferred to other wards following clinical improvement, and subsequent laboratory measurements were no longer available under the original inpatient case number. For the present analyses, only weekly blood samples were available and used. Following parameters were assessed: hemoglobin, thrombocytes, leukocytes, AST, ALT, CK, sodium, potassium, and phosphate. The reference ranges used for each laboratory parameter are displayed in Table 1.

*Illness duration.* Duration of illness (in years) was rated by patients’ therapists.

### Data analyses

Data were analyzed with *R* version 4.5.0 [28] in RStudio version 2026.01.0 + 392. The data and code with which all results can be reproduced can be accessed at 10.17605/OSF.IO/VTSF4. Descriptive statistics were computed using the *summarytools* package version 1.1.4. Analyses were performed using mixed-effects models, which allowed the inclusion of all available observations without requiring complete laboratory measurements at every assessment. For each laboratory parameter, values were categorized as below, within, or above the predefined clinical reference range, and the corresponding proportions were calculated for week 1 and week 6 (excluding missing values). To assess whether the proportion of values within the normal range changed between week 1 and week 6, generalized linear mixed-effects models with a binomial distribution and logit link were fitted for each laboratory parameter. Normal-range status (within vs. outside the reference range) was included as the dependent variable, week as a fixed effect, and participant ID as a random intercept to account for repeated measurements. The resulting *p*-values for the effect of week are reported.

To examine longitudinal changes in BMI and continuous laboratory parameters (hemoglobin, thrombocytes, leukocytes, AST, ALT, CK, sodium, potassium, and phosphate) over six weeks (i.e., across six time points), robust linear mixed-effects models were fitted using the *robustlmm* package version 3.3.3 [29]. Because assumptions underlying general linear models are often violated in clinical data, robust estimation approaches are recommended [30]. Time was treated as a continuous predictor, and models using orthogonal polynomial terms were fitted to capture both linear and quadratic trajectories over the six weeks [31]. To examine whether changes in BMI or laboratory parameters over the six-week treatment period differed depending on baseline BMI or illness duration, separate models were specified that included the interaction terms between time (linear and quadratic polynomial components) and the respective moderator (BMI at admission or illness duration). In addition, a sensitivity analysis was conducted by re-estimating all illness-duration models with BMI at admission included as a covariate. Significant interaction effects were probed with the *interactions* package version 1.2.0.

*P*-values were obtained by combining the robust *t*-values with Satterthwaite-approximated degrees of freedom based on non-robust mixed models computed with the *lme4* package version 1.1.37 [32]. Given the large sample size, effects were considered significant when *p* ≤. 005 [33]. To assess the robustness of the findings, sensitivity analyses were performed in a subgroup of patients who achieved the predefined minimum treatment target weekly weight gain of ≥ 0.70 kg. Average weekly weight gain was calculated based on weight measurements obtained at week 1 and week 6. The same statistical models as in the primary analyses were applied to this subgroup. Detailed results of the sensitivity analysis are presented in the Supplementary Material.

## Results

### Laboratory parameters at admission and over the course of treatment

**Fig. 1:**
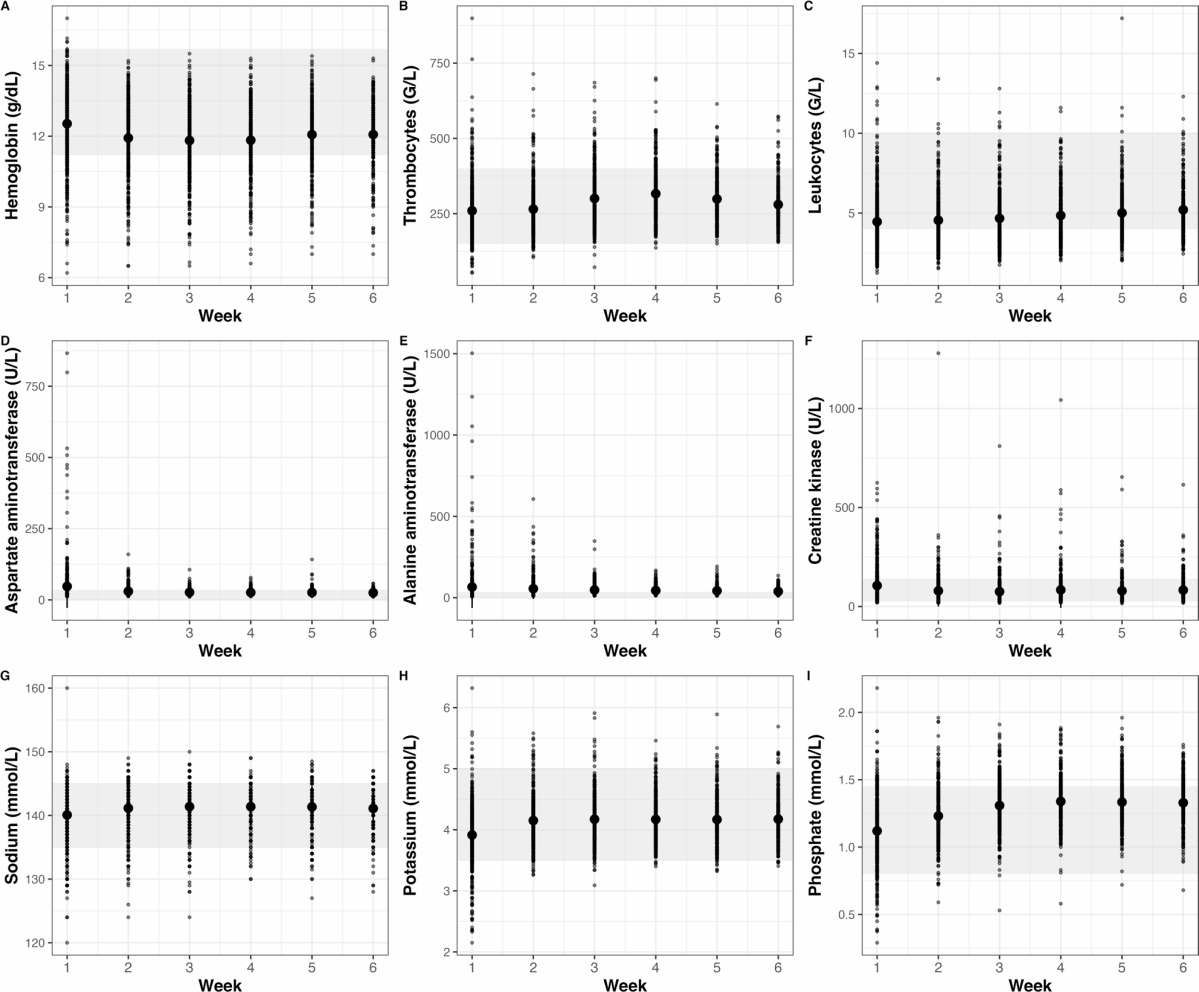
Scatter plots of **A** hemoglobin, **B** thrombocytes, **C** leukocytes, **D** aspartate aminotransferase, **E** alanine aminotransferase, **F** creatine kinase, **G** sodium, **H** potassium, and **I** phosphate levels during the six treatment weeks. Black dots represent individual observations, and larger black dots represent the arithmetic mean. Grey-shaded areas indicate the respective reference ranges

In the first week of specialized care, the laboratory values of many patients were outside the reference range (Fig. 1). Across all laboratory parameters, leukopenia, elevated transaminases, anemia, and elevated CK levels exhibited the highest proportion of values outside the clinical reference range at admission (Table 1). After six weeks of high-calorie refeeding with prophylactic phosphate supplementation initiated from day 1, hemoglobin, leukocytes, and ALT showed the highest proportion of values outside the clinical reference range. Electrolyte disturbances after six weeks were infrequent, with six patients (2.24%) showing hypokalemia and only one patient (0.38%) hypophosphatemia. All electrolyte abnormalities were mild, remained close to the lower reference range, and were not preceded by abnormal values in the previous week. In general, the prevalence of abnormalities in thrombocyte count, leukocyte count, AST, CK, sodium, potassium, and phosphate levels decreased significantly over time. However, in a sensitivity analysis restricted to patients who achieved the predefined weight gain target, the decrease in CK abnormalities was no longer statistically significant (Supplementary Material Table S1 & Figure S1).

**Table 1:** Proportions of patients with laboratory values below, within or above reference range in week 1 and week 6 with *p*-values for the time effect assessing changes toward normalization

Laboratory parameter	Reference range	Week 1 (*n* (%))				Week 6 (*n* (%))			
		Below reference	Within reference	Above reference		Below reference	Within reference	Above reference	Time effect *p*-value
Hemoglobin	11.2–15.7 g/dL	98 (18.9)	417 (80.3)	4 (0.77)		54 (20.3)	212 (79.7)	0 (0.00)	.849
Thrombocytes	150–400 G/L	37 (7.13)	447 (86.1)	35 (6.74)		0 (0.00)	246 (92.5)	20 (7.52)	< .001
Leukocytes	4.00–10.0 G/L	248 (47.8)	263 (50.7)	8 (1.54)		63 (23.7)	200 (75.2)	3 (1.13)	< .001
Aspartate aminotransferase	0.00–35.0 U /L	0 (0.00)	329 (63.4)	190 (36.6)		0 (0.00)	243 (90.7)	25 (9.33)	< .001
Alanine aminotransferase	0.00–35.0 U /L	0 (0.00)	289 (55.7)	230 (44.3)		0 (0.00)	143 (53.4)	125 (46.6)	.702
Creatine kinase	26.0–140 U/L	6 (1.16)	422 (81.3)	91 (17.5)		3 (1.13)	229 (86.1)	34 (12.8)	< .001
Sodium	135–145 mmol/L	44 (8.48)	449 (86.5)	26 (5.01)		10 (3.73)	247 (92.2)	11 (4.10)	< .001
Potassium	3.50–5.00 mmol/L	78 (15.0)	432 (83.2)	9 (1.73)		6 (2.24)	254 (94.8)	8 (2.99)	< .001
Phosphate	0.80–1.45 mmol/L	43 (8.29)	432 (83.2)	44 (8.48)		1 (0.38)	199 (74.5)	67 (25.1)	.004

**Fig. 2:**
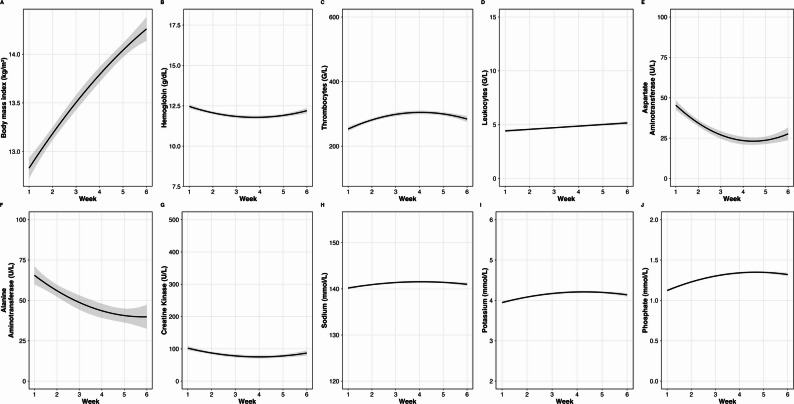
Model-estimated trajectories of **A** body mass index, **B** hemoglobin, **C** thrombocytes, **D** leukocytes, **E** aspartate aminotransferase, **F** alanine aminotransferase, **G** creatine kinase, **H** sodium, **I** potassium, and **J** phosphate across the six-week treatment period. Solid lines represent predicted values derived from mixed-effects models, and shaded areas indicate 95% confidence intervals. Second-order polynomial trajectories were specified for all parameters except leukocytes, which were modeled using a linear term

Across the six-week treatment period, most dependent variables followed non-linear trajectories (Fig. 2). BMI increased non-linearly across the six measurement points, with a steeper increase at the beginning of treatment and leveling off after six weeks (effect of time^2^: *b* = −1.35, *SE* = 0.23, *p* < .001; Fig. 2A). At week 6, the mean BMI was 14.3 kg/m² (*SD* = 1.31, range = 10.1–18.5), corresponding to a mean increase of 1.49 kg/m² (*SD* = 0.55) in the six-week treatment period. This BMI change corresponded to a mean weight gain of 4.02 kg (*SD* = 1.24), resulting in an average weekly weight gain of 0.80 kg/week (*SD* = 0.28, range = −0.67 to 1.89). At discharge, the mean BMI was 15.6 kg/m² (*SD* = 2.39, range = 9.59–23.5). The sensitivity analysis restricted to weight gain target achievers yielded largely comparable trajectory patterns. As expected, weight restoration was greater in this subgroup, with a mean BMI increase of 1.75 kg/m² (*SD* = 0.43), corresponding to a mean weight gain of 4.70 kg (*SD* = 1.07) and an average weekly weight gain of 0.94 kg/week. Mean BMI was 14.3 kg/m² (*SD* = 1.38) at week 6 and 16.5 kg/m² (*SD* = 2.51) at discharge.

Hemoglobin (effect of time^2^: *b* = 10.19, *SE* = 0.72, *p* < .001; Fig. 2B), AST (effect of time^2^: *b* = 54.94, *SE* = 7.35, *p* < .001; Fig. 2E), ALT (effect of time^2^: *b* = −77.6, *SE* = 14.3, *p* < .001; Fig. 2F), and CK (effect of time^2^: *b* = 280.8, *SE* = 23.2, *p* < .001; Fig. 2G) decreased non-linearly across the six weeks, with an initial decline followed by a subsequent stabilization. Thrombocytes (effect of time^2^: *b* = −695.9, SE = 51.2, *p* < .001, Fig. 2C), sodium (effect of time^2^: *b* = −17.2, *SE* = 2.23, *p* < .001, Fig. 2H), potassium (effect of time^2^: *b* = −2.75, *SE* = 0.33, *p* < .001, Fig. 2I), and phosphate (effect of time^2^: *b* = −2.08, *SE* = 0.15, *p* < .001, Fig. 2J) increased non-linearly across the six weeks, with a steeper increase at the beginning of treatment followed by a subsequent decline and stabilization. Leukocytes increased linearly across the six measurement points (effect of time: *b* = 18.5, *SE* = 0.88, *p* < .001; Fig. 2D). The sensitivity analyses produced results consistent with the primary analyses, confirming the robustness of the observed findings (Supplementary Material, Figure S2).

### Moderator effects of baseline BMI

**Fig. 3:**
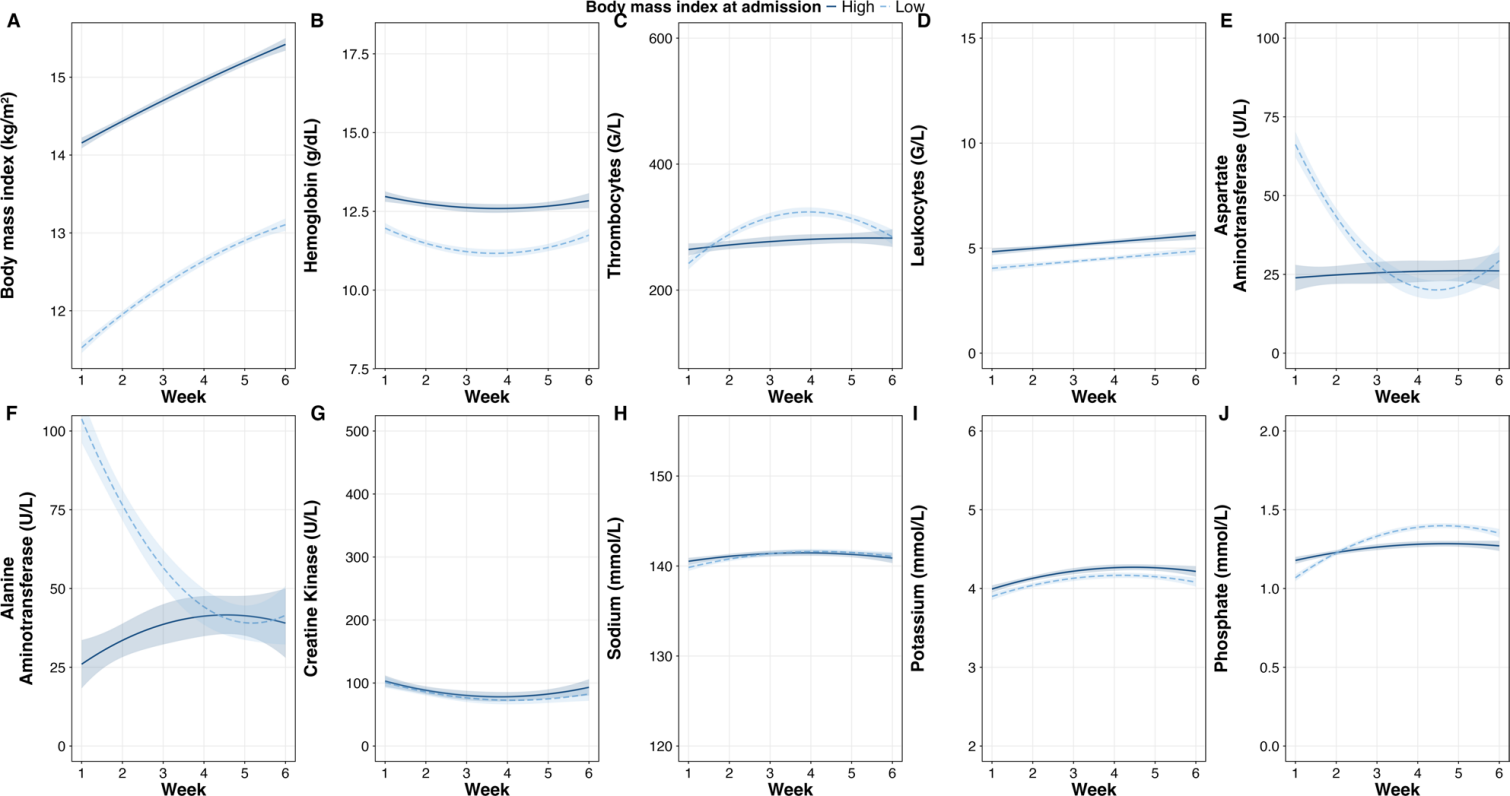
Simple slopes visualizing changes in **A** body mass index, **B** hemoglobin, **C** thrombocytes, **D** leukocytes, **E** aspartate aminotransferase, **F** alanine aminotransferase, **G** creatine kinase, **H** sodium, **I** potassium, and **J** phosphate across the six-week treatment period as a function of baseline body mass index. “High” and “low” refer to one standard deviation above and below the mean body mass index at admission. Grey-shaded areas represent 95% confidence intervals. Second-order polynomial trajectories were specified for all parameters except leukocytes, which were modeled using a linear term. Note that body mass index at admission did not moderate changes in leukocytes, creatine kinase, sodium, and potassium

BMI at admission moderated non-linear changes in BMI (interaction effect of time^2^ × baseline BMI: *b* = 0.68, *SE* = 0.17, *p* < .001), hemoglobin (interaction effect of time^2^ × baseline BMI: *b* = −3.30, *SE* = 0.53, *p* < .001), thrombocytes (interaction effect of time^2^ × baseline BMI: *b* = 397.3, *SE* = 37.1, *p* < .001), phosphate (interaction effect of time^2^ × baseline BMI: *b* = 0.83, *SE* = 0.11, *p* < .001), AST (interaction effect of time^2^ × baseline BMI: *b* = −33.6, *SE* = 5.49, *p* < .001), and ALT (interaction effect of time^2^ × baseline BMI: *b* = 0.83, *SE* = 0.11, *p* = .005). Specifically, lower baseline BMI was associated with a steeper increase in body weight (Fig. 3A), a more pronounced U-shaped trajectory of hemoglobin, characterized by a deeper nadir and a stronger curvature over time (Fig. 3B), a more pronounced inverted U-shaped pattern with levelling-off after six weeks in thrombocytes (Fig. 3C) and phosphate (Fig. 3J), and a more pronounced decrease and a later levelling-off of AST and ALT, whereas a higher BMI was associated with a more stable trajectory (Fig. 3E-F).

BMI at admission did not moderate non-linear changes in CK (*b* = −14.1, *SE* = 17.4, *p* = .420; Fig. 3G), sodium (*b* = 1.20, *SE* = 1.68, *p* = .470; Fig. 3H), and potassium (*b* = 0.27, *SE* = 0.25, *p* = .028; Fig. 3I). Linear change of leukocytes did not differ as a function of baseline BMI (interaction of time × baseline BMI: *b* = * -*0.89, *SE* = 0.65, *p* = .017; Fig. 3D). The sensitivity analyses produced results consistent with the primary analyses, confirming the robustness of the observed findings (Supplementary Material, Figure S3).

### Moderator effects of illness duration

**Fig. 4:**
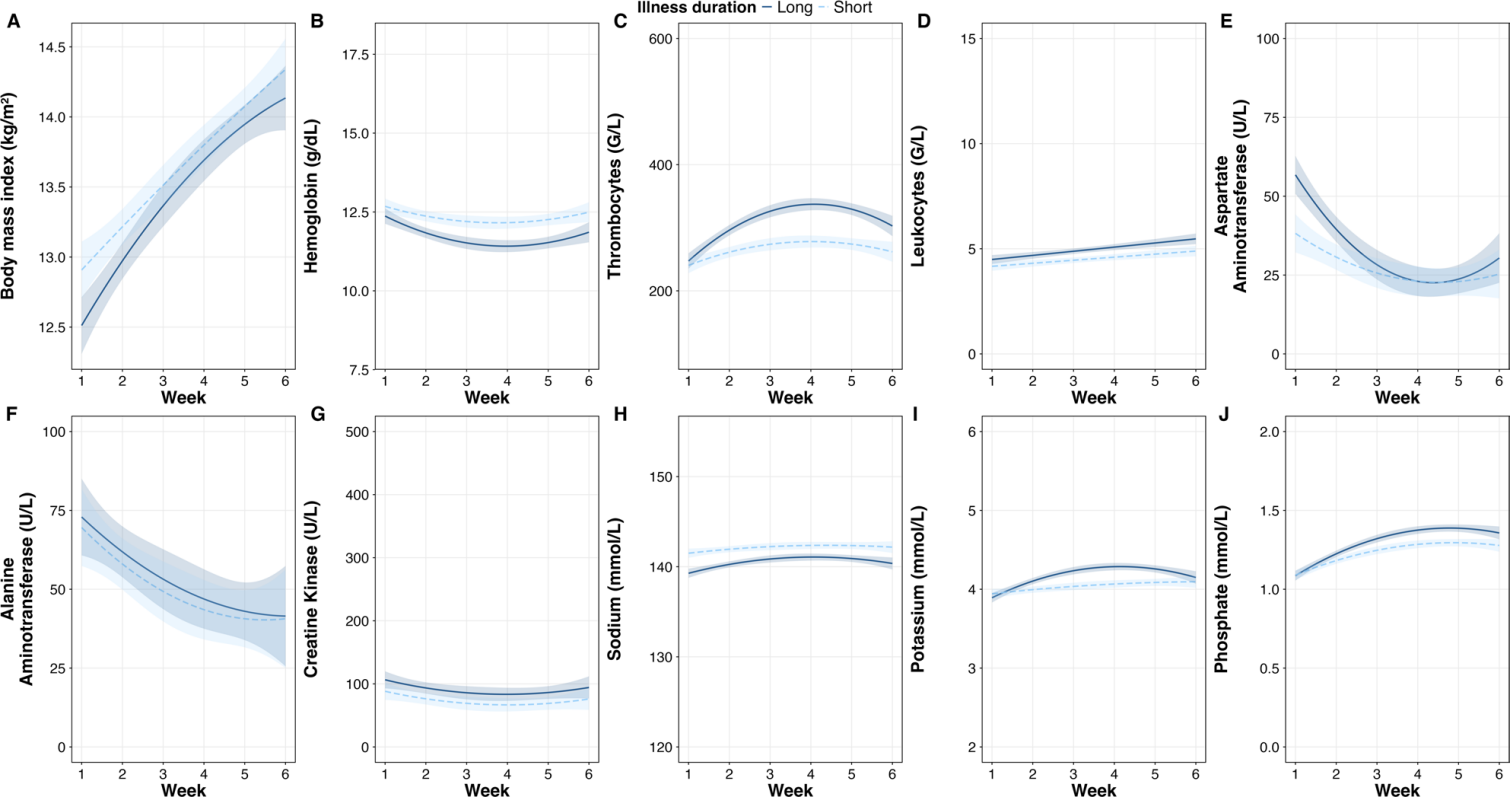
Simple slopes visualizing changes in **A** body mass index, **B** hemoglobin, **C** thrombocytes, **D** leukocytes, **E** aspartate aminotransferase, **F** alanine aminotransferase, **G** creatine kinase, **H** sodium, **I** potassium, and **J** phosphate across the six-week treatment period as a function of illness duration. “Long” and “short” refer to one standard deviation above and below the mean illness duration. Grey-shaded areas represent 95% confidence intervals. Second-order polynomial trajectories were specified for all parameters except leukocytes, which were modeled using a linear term. Note that illness duration did not moderate changes in alanine aminotransferase, sodium, and phosphate

Trajectories of several laboratory parameters and BMI differed by illness duration (Fig. 4). Illness duration moderated non-linear changes in BMI (interaction effect of time^2^ × illness duration: *b* = −0.10, *SE* = 0.03, *p* = .002), hemoglobin (interaction effect of time^2^ × illness duration: *b* = 0.40, *SE* = 0.12, *p* < .001), thrombocytes (interaction effect of time^2^ × illness duration: *b* = −29.6, *SE* = 7.80, *p* < .001), AST (interaction effect of time^2^ × illness duration: *b* = 3.05, *SE* = 1.03, *p* = .003), CK (interaction effect of time^2^ × illness duration: *b* = 10.6, *SE* = 3.01, *p* < .001), and potassium (*b* = −0.18, *SE* = 0.05, *p* < .001). Specifically, longer illness duration was associated with a flatter and earlier plateau of BMI increase over time (Fig. 4A), a more pronounced U-shaped trajectory in hemoglobin (Fig. 4B), a marked curvature and later levelling-off in thrombocytes and potassium (Fig. 4C and 4), a steeper initial decline followed by later stabilization in AST (Fig. 4E), and a more pronounced non-linear trajectory with higher overall levels of CK over time (Fig. 4G). Linear change of leukocytes did differ as a function of illness duration (interaction of time x illness duration: *b* = 0.43, *SE* = 0.12, *p* < .001; Fig. 4D). Specifically, longer illness duration was associated with a steeper linear increase. Non-linear changes in ALT, sodium, and phosphate did not differ as a function of illness duration (interaction of time^2^ x illness duration for all variables: ALT: *b* = −1.55, *SE* = 2.10, *p* = .046; sodium: *b* = −0.50, *SE* = 0.32, *p* = .012; phosphate: *b* = −0.04, *SE* = 0.02, *p* = .028, Fig. 4F, 4 and 4). Sensitivity analyses adjusting for admission BMI yield largely comparable results. However, an additional sensitivity analysis in the subgroup of weight gain target achievers demonstrated no significant illness duration-dependent differences in the trajectories of leukocytes, CK, or AST, while a significant association was observed for phosphate trajectories (Supplementary Material, Figure S4).

## Discussion

We examined how longitudinal trajectories of laboratory parameters changed during inpatient treatment in a specialized care unit for extreme AN with high-calorie refeeding initiated from day 1. During the six-week treatment period, many laboratory values stabilized, and the proportion of patients outside the clinical reference range decreased. Importantly, electrolyte disturbances were rare. This may indicate that high-calorie refeeding with routine prophylactic phosphate supplementation can be implemented safely under close medical monitoring. Baseline BMI and illness duration moderated the trajectories of BMI and several laboratory values. Lower BMI at admission was associated with more pronounced changes during refeeding, particularly during the early phase of treatment. In exploratory analyses, illness duration was associated with changes in several laboratory parameters as well, although these findings warrant cautious interpretation.

During the six-week treatment period, BMI increased rapidly during the initial phase and subsequently rose at a slower rate. This pattern is consistent with previous findings on inpatients with AN, which reported similar non-linear weight trajectories over an 18-week treatment period [24]. However, early BMI increases should be interpreted with caution, as weight changes during the initial phase of refeeding may partly reflect rehydration, glycogen repletion, and fluid shifts [34]. Because information on edema severity, eating disorder subtype, and volume status at admission were not available, the relative contribution of fluid shifts and tissue restoration cannot be determined. Nevertheless, patients gained an average of 4.0 kg during the six-week treatment period, corresponding to a mean weekly weight gain of 0.8 kg. Moreover, weight restoration continued beyond week 6, often after transfer from the acute medical unit to a specialized eating disorder treatment setting within the clinic. This sustained weight gain makes it unlikely that the observed increase in BMI was solely attributable to transient fluid retention. Notably, two-thirds of patients achieved the predefined weight gain target of 0.70 kg/week during the first six weeks. Together with the continued increase in BMI observed until discharge, these findings suggest that multidisciplinary inpatient treatment combining intensive medical monitoring, nutritional rehabilitation, and psychological support can promote substantial physiological recovery in patients presenting with extremely low BMI. Similar outcomes have been observed across other inpatient settings. More recently, a multicenter randomized controlled trial by Golden et al. [35] demonstrated that adolescents receiving a high-calorie refeeding protocol (starting at 2000 kcal/day) achieved earlier medical stabilization and had shorter hospital stays, without elevated safety risks or worse long-term outcomes. However, unlike the present study, routine prophylactic phosphate supplementation was part of our treatment protocol and should be considered when interpreting the laboratory trajectories.

### Trajectories of laboratory parameters during the six-week treatment period

The laboratory values in the six-week treatment period stabilized in many patients during refeeding. Importantly, sensitivity analyses restricted to patients who achieved the predefined minimum weekly weight gain target of 0.70 kg yielded largely comparable laboratory trajectories. This may suggest that the observed laboratory changes were not merely attributable to whether patients reached the predefined weight gain target and supports the robustness of the overall findings. Although mean hemoglobin levels showed a non-linear decline over time, the proportion of patients with anemia remained relatively stable across the treatment period. Interestingly, our observed anemia prevalence was only slightly higher than the 16.7% reported by De Filippo et al. [26] in a large outpatient sample with a higher mean BMI (15.9 kg/m^2^ vs. 12.6 kg/m^2^ in our sample) and slightly lower than the 21–39% range summarized by Hütter et al. [15]. This may indicate that anemia prevalence in persons with extreme AN is not higher than in persons with AN and higher BMI. Thrombocyte levels largely remained within the reference range, with only minor deviations, in line with previous studies [15]. Leukocyte levels improved over time, consistent with hematological recovery during nutritional rehabilitation [15]. Over six weeks, this proportion declined to about one-quarter, may indicate improvement with nutritional rehabilitation as previously mentioned. The observed improvement in hematological parameters likely reflects recovery of bone marrow function during nutritional rehabilitation.

Many patients had elevated ALT and AST levels. This finding is consistent with previous studies reporting abnormal liver enzymes in up to half of persons with AN [34]. While AST levels decreased significantly over time, the proportion of patients with elevated ALT remained largely unchanged. This discrepancy might reflect different underlying mechanisms and kinetics. Cuntz and Voderholzer [36] suggested that AST, being mostly mitochondrial, responds more rapidly to nutritional rehabilitation. ALT, mainly cytosolic, may remain elevated longer due to persistent subclinical hepatocellular stress [36]. Elevated ALT may warrant continued monitoring beyond acute refeeding phase. CK levels were frequently elevated at admission and showed improvement in the overall sample. However, this pattern was less pronounced in the subgroup of weight gain target achievers, suggesting considerable interindividual variability. Elevated CK levels have previously been linked to excessive physical activity in AN, although mechanisms related to severe malnutrition may also contribute [18].

Electrolyte levels are closely monitored during refeeding because of the risk of refeeding syndrome [18]. Previous studies on patients with extreme AN have reported substantial rates of hypophosphatemia [12, 13]. In the present study, under our structured high-calorie refeeding and prophylactic phosphate supplementation with close monitoring, hypophosphatemia was infrequent. Phosphate levels showed a shift toward higher values during the later treatment phase. The increase in hyperphosphatemia is likely attributable to routine phosphate supplementation in early refeeding to prevent refeeding hypophosphatemia, as supported by previous work [23]. Less than 10% of patients showed hypo- or hypernatremia during the six-week treatment period. In contrast, potassium deficiency was more common initially, but the levels stabilized over six weeks. This is consistent with prior findings that hypokalemia can resolve with rehydration and refeeding in inpatient care and minimization of purging behavior [18].

### Baseline BMI as moderator

The trajectory of several laboratory values was significantly shaped by patients’ nutritional status at admission to the inpatient care unit. Although several moderation effects reached statistical significance, not all translated into clinically relevant abnormalities. Therefore, these findings should primarily be interpreted as differences in the magnitude and trajectory of physiological adaptation during refeeding as a function of BMI at admission. Most notably, lower baseline BMI was associated with a steeper and more sustained BMI increase during treatment. AST was more elevated at admission and declined more steeply in patients with lower BMI. This aligns with earlier findings, which reported an association between underweight severity and liver enzyme abnormalities [36]. Baseline BMI moderated the trajectory of phosphate levels, with lower BMI being associated with a more pronounced change over time, potentially reflecting greater metabolic adaptation during refeeding and the influence of phosphate supplementation [23]. Other hematologic parameters, such as hemoglobin and thrombocytes, showed stronger and more pronounced non-linear changes in patients with lower BMI. This may reflect the greater extent of bone marrow suppression in patients with lower BMI, as malnutrition in AN has been shown to affect all hematopoietic lineages [34]. In contrast, leukocyte trajectories did not significantly differ as a function of baseline BMI. Importantly, sensitivity analyses restricted to patients who achieved the predefined weight gain target yielded largely consistent results, supporting the robustness of these findings.

### Illness duration as moderator

Illness duration was associated with differences in several physiological trajectories during the six-week treatment period. Longer illness duration was associated with a flatter BMI increase and earlier plateau during treatment. In addition, patients with a longer illness duration exhibited more pronounced nonlinear trajectories in several laboratory parameters, including hemoglobin, thrombocytes, potassium, CK, and AST levels. These findings are broadly consistent with previous studies reporting associations between longer illness duration and hematological abnormalities, as well as lower potassium levels [26, 27]. However, the underlying mechanisms remain unclear. Stronger fluctuations in hemoglobin and thrombocytes may reflect more pronounced bone marrow suppression in chronically malnourished persons, as hematopoietic abnormalities affecting multiple cell lineages are well documented in AN [34]. Furthermore, prolonged undernutrition may be accompanied by adaptive physiological and metabolic responses [37, 38]. Interestingly, illness duration was associated with AST but not with ALT trajectories. Given that AST is present not only in the liver but also in skeletal muscle and other tissues, whereas ALT is more liver-specific, this finding may indicate that the observed association is not exclusively related to hepatic function. However, the mechanisms underlying this differential pattern remain unclear and should not be overinterpreted. Sensitivity analyses additionally adjusted for admission BMI yielded largely comparable results, suggesting that the observed associations were not solely attributable to differences in baseline BMI. However, some moderation effects were less consistent in analyses restricted to patients who achieved the predefined weight gain target. In general, findings related to illness duration should be interpreted cautiously.

### Limitations

Several limitations should be acknowledged. The findings are based exclusively on a sample of female inpatients in a specialized unit with AN and a BMI lower than 15 kg/m^2^. Therefore, the results may not generalize to people with milder forms of AN, males, or patients treated in less intensive or outpatient settings. Information on AN subtype was not available in the present dataset. Previous research suggests subtype-specific laboratory and electrolyte profiles [39]. Therefore, potential differences could not be examined. All patients received care within a structured and highly monitored refeeding program with prophylactic phosphate supplementation, which may have influenced laboratory trajectories, particularly for parameters sensitive to supplementation. This was an observational study reflecting routine clinical practice. Consequently, causal conclusions regarding the effects of the refeeding protocol cannot be drawn. Furthermore, prophylactic phosphate supplementation was routinely administered during early refeeding. Individual adherence to phosphate supplementation was not systematically documented. Consequently, serum phosphate concentrations may not fully reflect the natural course of phosphate homeostasis during nutritional rehabilitation, and the incidence of hypophosphatemia may have been influenced by clinical management. Illness duration was based on retrospective self-report and was subject to substantial missingness. This limits the interpretability of moderation effects and introduces potential bias. Furthermore, illness duration does not necessarily correspond to the duration of severity of undernutrition and should therefore be interpreted as a proxy of illness chronicity rather than a direct measure of starvation exposure. The results regarding illness duration should be interpreted with caution.

Although linear and generalized mixed-effects models allowed the inclusion of all available observations without requiring complete laboratory measurements at every assessment, laboratory data were incomplete at week 6. Consequently, selection bias cannot be excluded, and the estimated laboratory trajectories may have been affected by this. Another limitation is the lack of a systematic assessment of hypoglycemia during hospitalization. Although hypoglycemia represents an important manifestation of medical instability in patients with extreme AN and may contribute to life-threatening complications, systematic glucose monitoring data were not available for the present analysis. Likewise, systematic data on fluid status, edema, purging behaviors, intravenous fluid administration, and actual caloric intake were not available and could therefore not be included in the analyses. Although meal completion was closely supervised and missed calories were routinely replaced by nutritional supplements, individual differences in nutritional exposure during treatment could not be directly quantified. Furthermore, early BMI changes may partly reflect fluid shifts rather than exclusively tissue restoration. Finally, ferritin and other iron status markers were not available for this study. Therefore, the etiology of anemia could not be further characterized, and differentiation between iron deficiency and anemia related to severe malnutrition was not possible.

### Future directions

Future studies should aim to replicate these findings in more diverse populations, including men. Expanding the observation period beyond six weeks would allow a better understanding of long-term normalization processes and the persistence of laboratory abnormalities. Future research should also incorporate more detailed assessments of actual caloric intake and nutritional exposure during refeeding to better disentangle the effects of nutritional rehabilitation from individual differences in treatment implementation. Furthermore, systematic assessment of body composition, edema, and fluid status would help clarify the extent to which early weight gain reflects tissue restoration versus transient fluid shifts. Given the clinical importance of hypoglycemia in severely underweight patients, standardized glucose monitoring during refeeding should also be considered. Finally, future studies should investigate whether the duration of severe undernutrition or critical low BMI is more strongly associated with laboratory trajectories than overall illness duration. Given the high metabolic vulnerability of extremely underweight persons and the effectiveness of high-calorie refeeding, it would be interesting to investigate patients’ perspectives on refeeding before and after their treatment. This could inform communication strategies during refeeding and potentially reduce treatment discontinuation.

## Conclusion

In conclusion, this study provides detailed longitudinal data on laboratory trajectories during the first six weeks of inpatient nutritional rehabilitation with prophylactic phosphate supplementation in patients with severe and extreme AN with a BMI under 13 kg/m^2^ at admission. The observed trajectories support previous evidence suggesting that high-calorie refeeding initiated from the first day of admission, when combined with routine prophylactic phosphate supplementation and close medical monitoring, may be implemented safely in a specialized inpatient setting and may promote rapid stabilization and normalization of laboratory parameters. These findings may support clinical decision-making by providing a characterization of the expected course of laboratory values during early refeeding and underscore the importance of close medical and laboratory monitoring during this dynamic phase of treatment.

## Supplementary Information

Below is the link to the electronic supplementary material.

Supplementary Material 1.

## Data Availability

The data and code with which all results can be reproduced can be assessed at 10.17605/OSF.IO/VTSF4.

## Abbreviations

AN: Anorexia nervosa
ALT: Alanine aminotransferase
AST: Aspartate aminotransferase
BMI: Body mass index
CK: Creatine kinase
