# Supplementary material for: Trajectories of blood parameters and body weight during inpatient treatment of anorexia nervosa with extreme underweight
Source: J Eat Disord. 2026 Aug 12;14:186. doi: 10.1186/s40337-026-01734-3 (PMC13471363; doi:10.1186/s40337-026-01734-3)
Supplement: Supplementary file 1 [file 40337_2026_1734_MOESM1_ESM.docx]

**Supplementary Material**

This supplementary material provides details of the sensitivity analyses performed in the subgroup of patients who achieved the predefined minimum target weight gain of ≥0.7kg/week. Average weekly weight gain was calculated based on weight measurements obtained at week 1 and week 6. Inclusion in the sensitivity analyses required available weight data at both time points. Of the full sample (N = 519), 142 participants (27.4%) were excluded due to missing data at week 6. Consequently, 377 participants were eligible for the calculation of average weekly weight gain. Among these participants, 250 (66.3%) achieved the minimum target, while 127 (33.7%) did not.

**Table S1.** *Proportions of patients with laboratory values below, within or above reference range in week 1 and week 6 of “target achievers” (n = 250) with p-values for the time effect assessing changes toward normalization*

| Laboratory Parameter | Reference range | Week 1 (*n* (%)) | | |  | Week 6 (*n* (%)) | | | Time-effect |
| --- | --- | --- | --- | --- | --- | --- | --- | --- | --- |
|  |  | Below  reference | Within  reference | Above  reference |  | Below  reference | Within reference | Above reference | *p*-value |
| Hemoglobin | 11.2–15.7 g/dL | 37  (14.8) | 211  (84.4) | 2  (0.80) |  | 37  (20.2) | 146  (79.8) | 0  (0.00) | .193 |
| Thrombocytes | 150–400 G/L | 26  (10.4) | 211  (84.4) | 13  (5.20) |  | 0  (0.00) | 169  (92.3) | 14  (7.65) | < .001 |
| Leukocytes | 4.00–10.0 G/L | 124  (49.6) | 125  (50.0) | 1  (0.40) |  | 42  (23.0) | 139  (76.0) | 2  (1.09) | < .001 |
| Aspartate aminotransferase | 0.00–35.0 U / L | 0  (0.00) | 161  (64.4) | 89  (35.6) |  | 0  (0.00) | 169  (92.3) | 14  (7.65) | < .001 |
| Alanine aminotransferase | 0.00–35.0 U / L | 0  (0.00) | 129  (51.6) | 121  (48.4) |  | 0  (0.00) | 95  (51.9) | 88  (48.1) | .894 |
| Creatine kinase | 26.0–140 U/L | 4  (1.60) | 206  (82.4) | 40  (16.0) |  | 3  (1.65) | 137  (88.4) | 21  (11.5) | .010 |
| Sodium | 135–145 mmol/L | 23  (9.20) | 216  (86.4) | 11  (4.40) |  | 8  (4.35) | 168  (91.3) | 8  (4.35) | .004 |
| Potassium | 3.50–5.00 mmol/L | 42  (16.8) | 205  (82.0) | 3  (1.20) |  | 5  (2.73) | 172  (94.0) | 6  (3.28) | < .001 |
| Phosphate | 0.80–1.45 mmol/L | 30  (12.0) | 206  (82.4) | 14  (5.60) |  | 0  (0.00) | 128  (69.9) | 55  (30.1) | p = .002 |

**Figure S1.** Scatter plots of (A) hemoglobin, (B) thrombocytes, (C) leukocytes, (D) aspartate aminotransferase, (E) alanine aminotransferase, (F) creatine kinase, (G) sodium, (H) potassium, and (I) phosphate values in the six treatment weeks. Black dots represent individual observations and larger black dots represent the arithmetic mean. Grey-shaded areas indicate the respective reference ranges.

**Figure S2.** Model-estimated trajectories of (A) body mass index, (B) hemoglobin, (C) thrombocytes, (D) leukocytes, (E) aspartate aminotransferase, (F) alanine aminotransferase, (G) creatine kinase, (H) sodium, (I) potassium, (J) phosphate across the six-week treatment period. Solid lines represent predicted values derived from mixed-effects models, and shaded areas indicate 95% confidence intervals. Second-order polynomial trajectories were specified for all parameters except leukocytes, which were modeled using a linear term.

**Figure S3**. Simple slopes visualizing changes in (A) body mass index, (B) hemoglobin, (C) thrombocytes, (D) leukocytes, (E) aspartate aminotransferase, (F) alanine aminotransferase, (G) creatine kinase, (H) sodium, (I) potassium, (J) phosphate across the six-week treatment period as a function of baseline body mass index. “High” and “low” refer to one standard deviation above and below the mean body mass index at admission. Grey-shaded areas represent 95% confidence intervals. Second-order polynomial trajectories were specified for all parameters except leukocytes, which were modeled using a linear term. Note that body mass index at admission did not moderate changes in leukocytes, creatine kinase, sodium, and potassium.

**Figure S4**. Simple slopes visualizing changes in (A) body mass index, (B) hemoglobin, (C) thrombocytes, (D) leukocytes, (E) aspartate aminotransferase, (F) alanine aminotransferase, (G) creatine kinase, (H) sodium, (I) potassium, (J) phosphate across the six-week treatment period as a function of illness duration. “Long” and “short” refer to one standard deviation above and below the mean illness duration. Grey-shaded areas represent 95% confidence intervals. Second-order polynomial trajectories were specified for all parameters except leukocytes, which were modeled using a linear term. Illness duration significantly moderated trajectories of panel A body mass index, panel B hemoglobin, panel C thrombocytes, panel D leukocytes, panel E aspartate aminotransferase, panel G creatine kinase, and panel I potassium. Note that illness duration did not moderate changes in leukocytes, creatine kinase, aspartate aminotransferase, alanine aminotransferase, and sodium.
